# The ^125^Te Chemical Shift of Diphenyl Ditelluride: Chasing Conformers over a Flat Energy Surface

**DOI:** 10.3390/molecules24071250

**Published:** 2019-03-30

**Authors:** Marco Bortoli, Marco Dalla Tiezza, Cecilia Muraro, Giacomo Saielli, Laura Orian

**Affiliations:** 1 Dipartimento di Scienze Chimiche, Università degli Studi di Padova, Via Marzolo, 1-35131 Padova, Italy; marco.bortoli@studenti.unipd.it (M.B.); marco.dallatiezza@studenti.unipd.it (M.D.T.); cecilia.muraro@studenti.unipd.it (C.M.); giacomo.saielli@unipd.it (G.S.); 2 CNR, Istituto per la Tecnologia delle Membrane, Unità di Padova, Via Marzolo, 1-35131 Padova, Italy

**Keywords:** diphenyl ditelluride, ^125^Te chemical shift, relativistic DFT calculations, GAFF, MD simulations, NMR calculations

## Abstract

The interest in diphenyl ditelluride (Ph_2_Te_2_) is related to its strict analogy to diphenyl diselenide (Ph_2_Se_2_), whose capacity to reduce organic peroxides is largely exploited in catalysis and green chemistry. Since the latter is also a promising candidate as an antioxidant drug and mimic of the ubiquitous enzyme glutathione peroxidase (GPx), the use of organotellurides in medicinal chemistry is gaining importance, despite the fact that tellurium has no recognized biological role and its toxicity must be cautiously pondered. Both Ph_2_Se_2_ and Ph_2_Te_2_ exhibit significant conformational freedom due to the softness of the inter-chalcogen and carbon–chalcogen bonds, preventing the existence of a unique structure in solution. Therefore, the accurate calculation of the NMR chemical shifts of these flexible molecules is not trivial. In this study, a detailed structural analysis of Ph_2_Te_2_ is carried out using a computational approach combining classical molecular dynamics and relativistic density functional theory methods. The goal is to establish how structural changes affect the electronic structure of diphenyl ditelluride, particularly the ^125^Te chemical shift.

## 1. Introduction 

Diphenyl ditelluride (Ph_2_Te_2_) was first synthesized in 1894 [1,2,3]. Since then, the study on organotellurides has been rapidly increasing. In contrast to the historical notion that compounds of tellurium are difficult to handle and very sensitive to air and light, besides having a very pungent odor, many of these substances proved stable to air and light and were odorless [4,5]. Like organoselenides, organotellurides are currently widely used in organic synthesis to obtain useful intermediates, e.g., via transmetallation or cross-coupling reactions, and in the interconversion of functional groups [6,7,8]. In particular, Ph_2_Te_2_ proved to be efficient in catalytic oxidations of olefins [9]. In this reaction, the active catalyst is phenyl tellurol which forms upon Te–Te bond cleavage and can be oxidized to phenyltellurenic and phenyltellurinic acid. In fact, the chalcogen–chalcogen bond is soft and polarizable and can be easily broken [10], much more easily after initial oxidation, as reported for diphenyl diselenide (Ph_2_Se_2_) [11,12].

By analogy to Ph_2_Se_2_, Ph_2_Te_2_ has also been considered for its antioxidant potential, mimicking the enzymatic activity of glutathione peroxidase (GPx) [13,14]. Rocha and co-workers have reported evidence of thiol peroxidase activity of diphenyl ditelluride in mice [15,16]. The GPx-like mechanism of peroxide degradation was assessed in vitro with different thiols as reducing agents. Differently from what was found in the case of analogous diselenides—for which the active species is regarded to be a selenol/selenolate—in presence of tellurium the reaction seems to be associated with reversible changes in the chalcogen’s oxidation state [15]. Mechanistic differences in the oxidation step of GPx enzymatic cycle [17,18] have been recently elucidated in silico also by modeling the catalytic pocket of Se-GPx and of its Te-mutant [19].

Interesting studies about the toxicity of Ph_2_Te_2_ are available in the literature [20,21,22,23,24]. However, the toxicological data on Ph_2_Te_2_ and organotellurium compounds, in general, are not exhaustive and conflicting results were obtained, the majority of which lean towards the view that organotellurides have a higher toxicity compared to their selenium analogs [25]. 

**Scheme 1:**
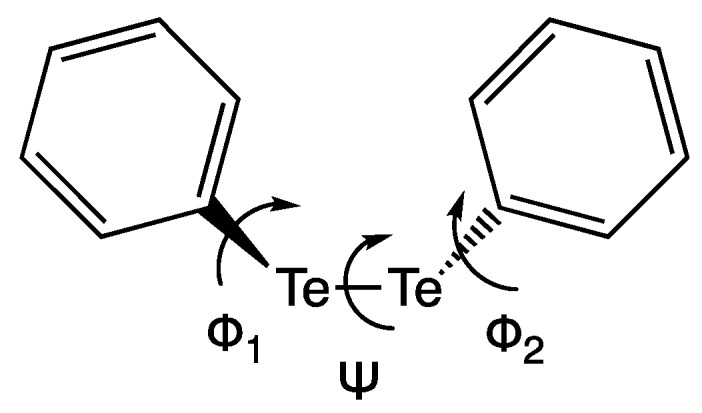
Diphenyl ditelluride; the C–Te–Te–C (Ψ) and C–C–Te–Te (Φ_1_ and Φ_2_) dihedral angles are shown.

Ph_2_Te_2_ is a chiroptical molecule, due to its peculiar structural features (Scheme 1). The dihedral angle Ψ has an equilibrium value of −89°, close to the value of H_2_O_2_; conversely, the absolute values of the dihedral angles Φ_1,2_ may be both close to 0° or both close to 90°, defining the orientation of the phenyl rings in two minimum energy geometries, respectively. In the crystallographic structure [26], the values of the Φ_1,2_ dihedral angles are 96° and 90°. This arrangement is found to be lower in energy, as revealed by accurate calculations at different levels of theory [10]. The conformation with Φ_1,2_ ≈ 0 is another minimum, although lying very close in energy to the former one, as we will see below. Importantly, in solution the phenyl rings are almost free to rotate. The fast conformational motions of the phenyl rings were observed experimentally [27] as well as quantified through density functional theory (DFT) calculations [10,28]. The barrier in gas phase is about 5.5 kcal mol^−1^ for the rotation about Ψ and 0.9 kcal mol^−1^ for the rotation about Φ_1,2_ at B3LYP/cc-pVTZ(-PP) level of theory [25]. 

Nowadays, computational protocols based on DFT are widely used for the investigation of structural [29] and spectroscopic properties of organic and organometallic molecules [30]. In particular, the prediction of NMR chemical shifts has been the subject of several studies, especially concerning ^1^H and ^13^C chemical shifts of organic molecules [31,32,33], also for the case of natural substances including heavy atoms [34]. In the latter case, relativistic effects must be accounted for, because of the well-known HALA (heavy atom on light atom) effect [35,36]. Relativistic effects are even more important when the sought after NMR properties are those of the heavy atom itself [37], as is the case of ^125^Te-NMR properties in organotellurides.

^125^Te-NMR parameters have been the subject of computational works [38,39,40]. For example, Rusakova et al. recently investigated several organotellurides and organoditellurides using a full four-component DFT relativistic approach. They observed that a very good agreement between calculated and experimental data could be obtained after inclusion, besides relativistic effects, of solvent and vibrational contributions [41]. However, diphenyl ditelluride was not included in the set of studied compounds. 

In a recent paper [42], Elder and Vargas-Baca reported an experimental investigation of the aggregation behavior in solution of some derivatives of diphenyl ditelluride. They used ^125^Te-NMR spectroscopy, supported by relativistic DFT calculations. Based on the concentration dependence of the δ (^125^Te), the authors proposed that aggregation occurs in low polarity solvents, e.g., hexane, toluene, and dichloromethane. However, the observed variations were of the order of ≈ 10 ppm/mol L^−1^, that is varying the concentration from 10^−2^ M to 10^−1^ M, would roughly change the ^125^Te chemical shift of the investigated compounds by at most 1 ppm. The effect of the temperature, in the investigated range, was comparable, of the order of 0.2 ppm/K. Nevertheless, the authors stated in their Conclusions that “the influence of concentration on the measured δ^125^Te, however, is dwarfed by the effects of conformational changes or the dielectric properties of the medium” [42]. 

Therefore, in this work, we will analyze the issues related to the calculation of the chemical shift of ^125^Te of diphenyl ditelluride; emphasis will be given to the dependence of δ(^125^Te) on conformational effects and the difficulties related with an accurate modeling of the conformational energy landscape, this one being an essential requisite for a reliable prediction of NMR properties. By using purely quantum as well as combined classic and quantum chemistry methods, we discuss the factors affecting the results, that is the geometry and the electronic effects. Particularly, the analysis of the dependence of the paramagnetic component of the shielding constant on selected relevant geometrical parameters such as the Te–Te and Te–H bond lengths in a simpler model compound, provides general insight for the calculation of δ(^125^Te) in organotellurides. 

## 2. Results

**Figure 1:**
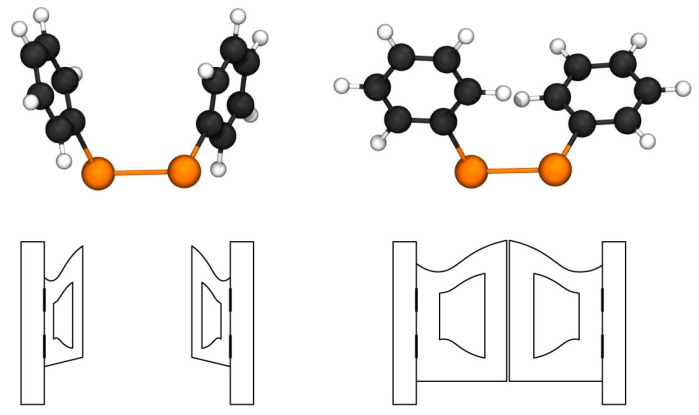
Fully optimized conformers of Ph_2_Te_2_, namely open (**left**) and closed (**right**); the corresponding open and closed doors are sketched below. Level of theory: ZORA-OPBE/TZ2P.

Recent computational studies on Ph_2_Te_2_ and its selenium analog [10,28] have revealed the presence of two stable minima. In fact, we have located two conformers on the diphenyl ditelluride potential energy surface (PES), displaying very similar values for the C–Te–Te–C dihedral (Ψ, −86° and −89°, respectively) but differing values of the C–C–Te–Te dihedrals (Φ_1,2_ = 100° and 10°, respectively). We refer to these two structures as the ‘open’ (Figure 1, left, with Φ_1,2_ dihedrals of 100°) and the ‘closed’ (Figure 1, right, with Φ_1,2_ dihedrals of 10°) conformer, due to the arrangement of the phenyl rings that resemble open and closed doors, respectively. They lie very close in energy as, at the ZORA-OPBE/TZ2P level of theory, the closed conformer is found only 0.5 kcal mol^−1^ above the open one, which is the most stable, in agreement with the crystallographic structure [26]. Both fully optimized structures were used for the calculation of the ^125^Te isotropic shielding constants (σ) (If not differently stated, a single value for the ^125^Te shielding constant/chemical shift is provided for Ph_2_Te_2_, which is the average of the almost identical values computed for each Te nucleus), which resulted in 2095.4 and 2390.2 ppm for the open and closed configurations, respectively, calculated in chloroform at the COSMO-ZORA-OPBE/QZ4Pae level of theory on the ZORA-OPBE/TZ2P optimized geometries (this level of theory will be referred to as COSMO-ZORA-OPBE/QZ4Pae//ZORA-OPBE/TZ2P); they correspond to δ(^125^Te) equal to 574.8 and 280.0 ppm, respectively, using Me_2_Te as a reference. These computed values reveal that the phenyl orientation strongly affects the ^125^Te chemical shift, as the difference amounts to 294.8 ppm. For a comparison with the experimental value of the δ(^125^Te) of Ph_2_Te_2_, which is 420.8 ppm [43], the Boltzmann-weighted mean with an open to closed ratio of 70/30 was computed, i.e., 486.4 ppm. The inclusion of spin orbit coupling [41,44] leads δ(^125^Te) of 476.8 and 184.5 ppm in the open and closed conformer, respectively (level of theory: COSMO-SO-ZORA-OPBE/QZ4Pae//ZORA-OPBE/TZ2P); the Boltzmann averaged δ(^125^Te) is 389.1 ppm. In the case of the open and closed conformers, for which the values of the C–Te–Te–C dihedral angle are similar, spin orbit effects can be regarded as a constant correction (see Figure S2). However, the spin orbit effect is significant, i.e., about 100 ppm, and it is due to HAHA (heavy atom on heavy atom) effect analogous to the HALA effect discussed in the Introduction: since the molecule of interest has two Te atoms and the reference, Me_2_Te only one, such effect on the shielding constant cannot be cancelled in the calculation of the chemical shift. Nonetheless, the most significant difficulty in the calculation of the δ(^125^Te) of Ph_2_Te_2_ is intrinsic in the nature of the molecular PES, being related to the small energy difference between the conformers. We note, in fact, that even an error as small as 0.1 kcal mol^−1^ in the calculation of the relative energy, affects the population ratio at room temperature by a factor of about 1.2. It is worth mentioning that calculations of the δ(^125^Te)—with a full four component relativistic method, also taking into account solvation effects—were performed for a series of mono tellurides in a recent paper [41]. The authors report a mean absolute error of 23 ppm for a series of six compounds. Taking into account the presence of two Te nuclei and the conformational issues, the inclusion of scalar relativistic effects for our system is sufficient and is not the most important error source.

With the aim of understanding the relation between the structure and the value of δ(^125^Te), a classical molecular dynamics (MD) simulation was employed to obtain a set of structures spanning the conformational space of Ph_2_Te_2_. The generalized AMBER force field (GAFF) parameters required to completely define our molecule were generated using a computational protocol recently devised by some of us (see Materials and Methods section for further details). We could not use the GAFF parameters of (Ph_2_Te_2_) reported in [28] because they were obtained using quantum mechanical calculations performed at a different level of theory, i.e., B3LYP/cc-pVTZ(-PP). As demonstrated in this work, different functionals lead to slightly different equilibrium bond lengths and angles, which have a strong impact on the calculations of the shielding constant of the ^125^Te nucleus. Thus, it is of fundamental importance that the force field parameters are derived consistently. 

**Figure 2:**
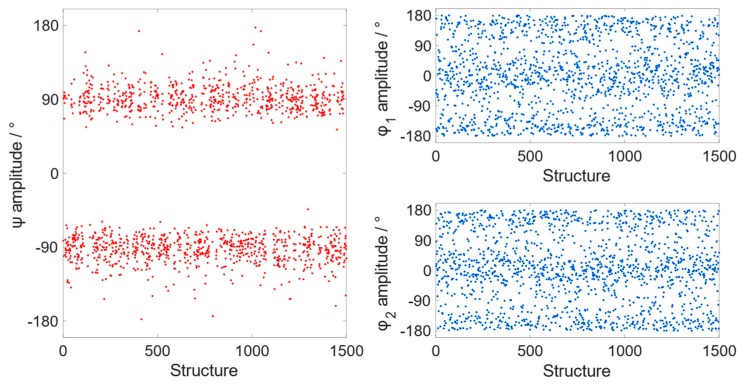
Values of the Ψ (**left**) and Φ_1,2_ (**right**) dihedral angles for the structures extracted from the MD simulation.

A 500 ns MD simulation on a single Ph_2_Te_2_ molecule, solvated with chloroform in a cubic box with a surrounding buffer of 12 Å, was carried out and 1500 structures were extracted at intervals of 300 ps. A structural analysis of the collected snapshots showed values of the Ψ dihedral angle clustered around 90° or −90° during the whole simulation (Figure 2), which are very close to the value obtained from the single molecule quantum mechanical optimization (−86° and −89° for the open and closed conformer, respectively). In contrast, the values of Φ_1,2_ dihedrals are more spread out, encompassing the whole 360° range. This is in agreement with the fact that Ψ and Φ have very different rotational barriers, estimated to be about 9 and 0.5 kcal mol^−1^ in gas phase at ZORA-OPBE/TZ2P [10]. During the MD simulation, the Te–Te bond distance fluctuates around a mean value of 2.67 Å, which is close to the average of the corresponding bond lengths in the open (2.69 Å) and the closed conformer (2.66 Å) optimized at the ZORA-OPBE/TZ2P level, with a standard deviation of 0.07 Å.

For each sampled snapshot, the ^125^Te isotropic shielding constants (σ) of both Te nuclei of the isolated Ph_2_Te_2_ molecule were calculated at the COSMO-ZORA-OPBE/QZ4Pae level of theory and averaged. The mean value of the calculated δ(^125^Te) was 501.9 ppm with a standard deviation of 269.5 ppm, clearly denoting that the δ(^125^Te) is highly sensitive to the geometrical structure. δ(^125^Te) values fall between a minimum of −225.5 ppm and a maximum of 1503.4 ppm (see Figure S1), an interval covering a good part of the known ^125^Te-NMR chemical shift range [40]. Interestingly, we have analyzed the ^125^Te shielding constants, also extracting a decreasing number of snapshots and found that the result does not change when using 1000 snapshots and the deviation remains smaller than 10 ppm up to an average based on 250 snapshots. Since, as noted above, the spin-orbit correction is non-negligible, we also calculated the mean value of the shielding constant using the spin-orbit relativistic Hamiltonian: the average δ(^125^Te) based on the previous 250 MD snapshots at the COSMO-SO-ZORA-OPBE/QZ4Pae level of theory is 403.8 ppm. This result is in very good agreement with the experimental value, being underestimated by only 17 ppm. 

A benchmark study was conducted to assess the effect of the functional and of the basis set on the geometry of the conformers and thus on δ(^125^Te) values. In a previous computational study by some of us [10], the focus was centered on the deviation of the computed Ph_2_Te_2_ geometry from the crystallographic structure, to assess the best method for geometry optimization. Therefore, the computations were carried out starting from the crystallographic conformer—i.e., the open one—which was identified correctly as a minimum using 25 different functionals. The conclusion was drawn that the choice of the functional is not so crucial to predict a fairly good minimum energy structure of Ph_2_Te_2_. Conversely, in the same study, differences among the methods arose when computing reaction energies. For homolytic Te–Te bond dissociation, only few functionals provide good agreement with experimental data—including OPBE, OLYP, and B3LYP—and among these ones only OLYP provides the correct dissociation energy trend when comparison is made between Ph_2_Se_2_ and Ph_2_Te_2_.

For our analysis, the structures of both conformers are needed to obtain δ(^125^Te) values close to the experimental one. Therefore, a systematic study on both conformers was carried out (i) to locate them on the PES testing different functionals and basis sets; and (ii) to evaluate the structural and energy discrepancies among them when changing the computational method. The structures of the closed and open conformers were fully optimized using 23 different functionals. The relative single point energies and the significant geometrical parameters, i.e., the Te-Te bond length (R) and the values of the Ψ/Φ_1,2_ dihedral angles are reported in Table 1.

Importantly, both conformers are found on the PES employing all the selected functionals and the energy differences between the open and closed structures are within a 1 kcal mol^−1^, except when LDA and M06-L are used, for which the open-closed energy difference amounts to −2.1 and −1.5 kcal mol^−1^, respectively. The Te–Te bond length (experimental value: 2.71 Å [26]) results systematically longer in the open conformer with all the tested functionals. However, the differences between these bond lengths in the two conformers is of hundredths of Å and changes slightly when changing functional, as it goes from a minimum of 2.67 Å in the case of the open structure and of 2.64 Å in the closed conformer when using OPBE0 to a maximum of 2.78 and 2.74 Å for the open and closed conformers when using BLYP. A peculiar behavior is found for the dihedral angle Ψ when looking at the results obtained with the functionals empirically corrected for dispersion. In these cases, Ψ is found to be appreciably smaller in the open conformer, resulting in structures in which the phenyl rings are almost stacked. Nevertheless, this geometrical difference does not significantly affect the energies of the conformers which are found to be very close to each other.

Additionally, full geometry optimizations of both conformers were carried out with the ZORA-OPBE functional combined with basis sets of different size to check whether the basis set choice may affect the final geometry. This test has confirmed once again the flatness of the PES of Ph_2_Te_2_, as with the smallest basis sets (i.e., SZ and DZP of single and double-ζ quality, respectively) the closed conformer was never located, but all attempts ended up in the open conformation. Increasing the number of the functions (i.e., employing the TZ2P and QZ4P basis sets) resulted in a more accurate description of the PES of Ph_2_Te_2_, which correctly returned both optimized structures although with minimal relative energy differences. 

These results indicate that the main issue to consider in order to calculate a δ(^125^Te) close to the experimental value is rooted in the accurate description of the molecular geometry and of the PES of Ph_2_Te_2_ and all functionals perform quite similarly. While we cannot act on the nature of the PES, it is desirable to understand how the calculated δ(^125^Te) is affected by changes in the relevant geometrical parameters, which are related not only to the different conformation, but more in general to even small differences on the optimized geometries provided by the different methods. In order to tackle this aspect, a decomposition analysis of the gas phase shielding constant was carried out (see the Materials and Methods section). The most important results come from the paramagnetic part of the shielding constant (σ_p_) as it is the one that displays the greatest variation upon geometrical modification of the structure. Moreover, for simplicity and in order to have a much broader view on the importance of the electronic environment of ^125^Te, the shielding constant analysis was first performed using a smaller model compound—i.e., PhTeH—focusing on the lengthening of the Te–H bond and on the rotation of the dihedral angle H–Te–C–C. 

**Figure 3:**
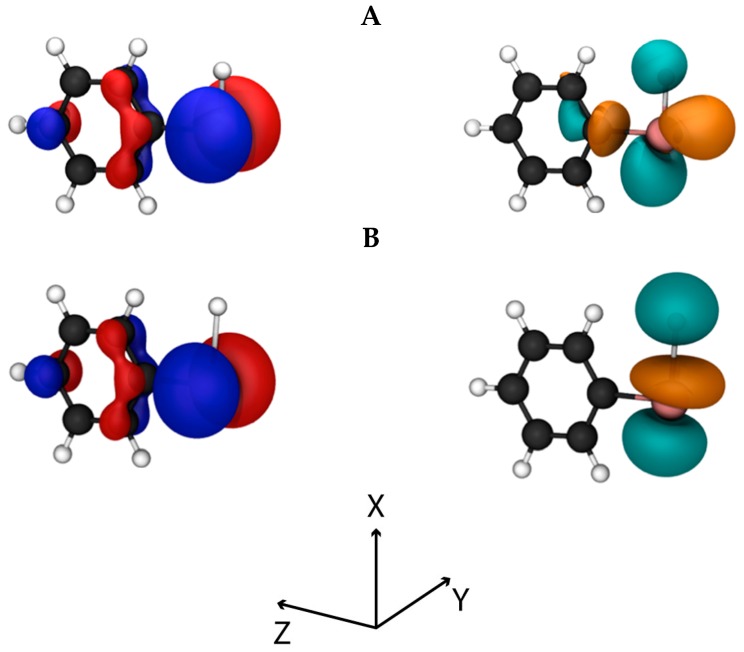
(**A**) HOMO (left) and LUMO (right) orbitals of PhTeH. (**B**) HOMO (left) and LUMO (right) orbitals of PhTeH at the maximum imposed deformation of the Te–H bond (2.66 Å). Isodensity 0.05 a.u. Level of theory: ZORA-OPBE/QZ4Pae//ZORA-OPBE/TZ2P.

We found that the total ^125^Te shielding constant in PhTeH (2646.7 ppm) is higher than those of both conformers of Ph_2_Te_2._, indicating that in the monochalcogenide the Te nucleus is more shielded. According to the scheme proposed by Schreckenbach et al. [45,46], we decomposed the isotropic shielding constant into its diamagnetic (σ_d_) and paramagnetic contributions (σ_p_), which amount to 5385.6 and −2738.9 ppm, respectively. σ_p_ can be expressed as the sum of two contributions, one arising from the coupling of pairs of occupied orbitals (σ_p_^occ-occ^) and the other from the coupling of an occupied and an empty orbital (σ_p_^occ-virt^) [46]. The interesting information comes from σ_p_^occ-virt^, since it is possible to discern which pairs of filled and empty molecular orbitals possess a strong magnetic coupling and thus contribute the most to the isotropic shielding tensor, that is to the chemical shift. This analysis showed that the sum of all the possible σ_p_^occ-virt^ terms is slowly converging to the total σ_p_. The decomposition of σ_p_ shows that the main term comes from the magnetic coupling of the HOMO–LUMO pair (about 32 % of the total σ_p_). The orbital analysis reveals that the HOMO is an antibonding orbital composed mainly of the Te p*_y_* orbital (Figure 3A, left), whereas the LUMO is also an antibonding orbital located in the *xz* plane resulting from a combination of the Te p_z_ and p_x_ orbitals, the p_x_ orbital on the carbon directly bonded to Te and the s orbital of the hydrogen atom linked to Te (Figure 3A, right). This composition is favorable, since after application of the magnetic coupling operator, the HOMO orbital is rotated along the *z*- or *x*- axis and can overlap substantially with the LUMO (see Materials and Methods).

**Figure 4:**
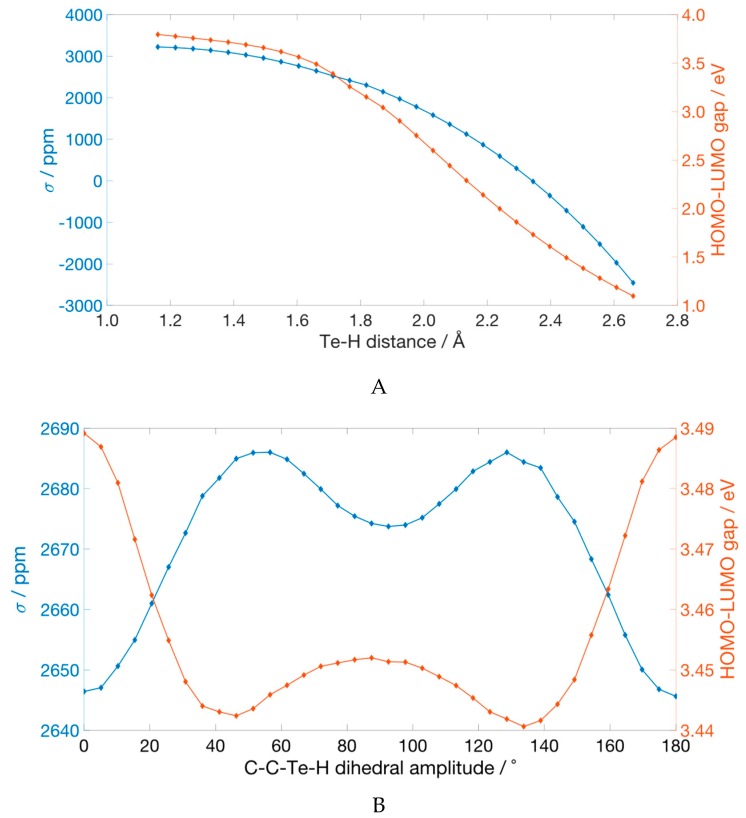
(**A**) Values of the ^125^Te isotropic shielding constant and of the HOMO-LUMO gap of PhTeH at different Te–H distances. (**B**) Values of the ^125^Te isotropic shielding constant of PhTeH at different values of the C–C–Te–H dihedral angle. Level of theory: ZORA-OPBE/QZ4Pae//ZORA-OPBE/TZ2P.

Upon stretching the Te–H bond, σ(^125^Te) decreases when going from the equilibrium value (1.66 Å) to 2.66 Å, i.e., the value changes from 2647.7 ppm to −2455.6 ppm (Figure 4A). This last value, when decomposed, results in a σ_d_ of 5389.6 ppm and a σ_p_ of −7843.2 ppm. Comparing these values to those of the equilibrium structure, it is clear that the paramagnetic part is responsible for the decrease of the σ(^125^Te). The decomposition of σ_p_ confirmed that the stretching of the Te-H bond enhances those terms arising from the occupied orbitals and the LUMO, as the energy of this anti-bonding orbital decreases with increasing Te–H distance (Figure 4A). Focusing on the largest contribution to σ_p_—i.e., the one arising from the HOMO–LUMO pair (Figure 3B), the term σ_p_^occ-virt^ changes from −891.4 ppm in the relaxed structure to −4998.8 ppm in the deformed geometry, while the energy gap between the two molecular orbitals shrinks from 3.49 to 1.10 eV.

The profile of the σ(^125^Te) as a function of the H–Te–C–C dihedral shows the presence of three minima at 0°, 92.6°, and 180° values of the dihedral angle (Figure 4B). The two structures at 0° and 180° are equivalent by symmetry and therefore have almost identical σ(^125^Te)—i.e., 2646.4 and 2645.6 ppm—whereas the relative minimum at 92.6° has a ^125^Te shielding constant value of 2673.7 ppm. The analysis of the σ_p_^occ-virt^ showed that when the dihedral angle H–Te–C–C is 0° or 180°, the empty molecular orbital mostly involved in the largest terms is the LUMO, which has the correct symmetry to allow favorable overlap, after rotation, with HOMO and HOMO-3 orbitals. As the dihedral value increases, the overlap decreases, resulting in a smaller—i.e., less negative—σ_p_. The relative minimum at 92.6° arises thanks to the presence of unfilled orbitals that have the correct symmetry to overlap with the HOMO after rotation around the Te–C bond, namely LUMO + 2 and LUMO + 3 (see Supplementary Materials Table S3). However, these unfilled orbitals lie at a higher energy than LUMO and the increased energy gap implies an overall less negative σ_p_, leading to a higher σ.

**Figure 5:**
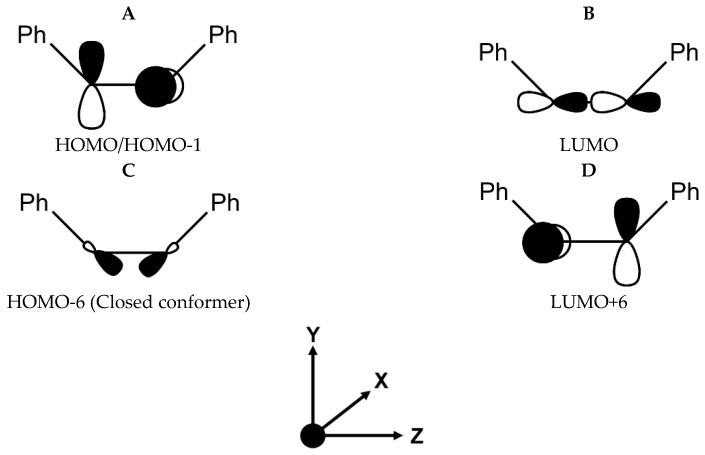
Most important inner and outer valence molecular orbitals (MOs) of Ph_2_Te_2_: HOMO/HOMO-1 (**A**), LUMO (**B**), HOMO-6 of the closed conformer (**C**) and LUMO+6 (**D**) Unless otherwise specified, the spatial part of the MOs is equal for both the conformers (open and closed).

For Ph_2_Te_2_, we decomposed the ^125^Te σ of both conformers into its diamagnetic and paramagnetic contributions. The results confirmed that σ_d_ changes only slightly for the two conformers—i.e., 5387.2 ppm for the closed conformer and 5388.5 ppm for the open one—and is almost identical to that computed for PhTeH, i.e., 5385.6 ppm. On the other hand, σ_p_ is appreciably different in the two conformers—i.e., −3495.1 ppm for the open one and −2990.7 ppm for the closed one. The decomposition of σ_p_ showed that the sum of all the σ_p_^occ-virt^ is slowly converging as for PhTeH. In addition, the multitude of terms makes it difficult to outline distinctive features that differentiate the chemical shifts. Therefore, we focused on the most relevant contributions. Particularly, we first addressed the σ_p_^occ-virt^ ascribed to the HOMO–LUMO pair, which has the smaller energy gap (3.05 eV in the closed conformer and 2.72 eV in the open conformer) and should therefore provide a substantial contribution to σ_p_. We found that in both conformers these terms are similar and represent approximatively 10% of the total σ_p_, i.e., −327.9 ppm (first Te nucleus) and −324.9 ppm (second Te nucleus) in the closed conformer, and −300.7 ppm (identical in each Te nucleus) in the open one. The HOMOs of both molecules display strong and either p*_x_* or p*_y_* atomic orbital character on the Te nuclei (Figure 5A). Conversely, the LUMOs have net Te p*_z_* atomic character. Either B*_y_* or B*_x_* field components will rotate the p*_x_* or p*_y_* to p*_z_*, thus leading to a non-null overlap integral with the LUMO. The component of the applied magnetic field is important for the evaluation of the integral in Equation (2) (see Materials and Methods): due to the mutually orthogonal nature of the p orbitals on the Te nuclei in the HOMO, the use of B*_y_* or B*_x_* determines a strong magnetic coupling only on one of the two chalcogens. 

The largest contribution to σ_p_ in the closed conformer comes from the HOMO-6–LUMO orbital pair (−994.7 ppm (first Te nucleus) and −992.4 ppm (second Te nucleus)), whereas for the open structure the orbital pair HOMO-1–LUMO + 6 gives the most substantial contribution (−707.4 ppm (first Te nucleus) and −707.2 ppm (second Te nucleus)). These orbital pairs are still significantly localized on Te nuclei with p atomic character (see Figure 5), but the origin of the strong magnetic coupling, despite their similar energy gaps, is different. In the closed conformer, the chalcogen nuclei have mixed p*_z_* and p*_x_*/p*_y_* character in the HOMO-6, these latter atomic contributions suitable for overlap with the Te p*_z_* based LUMO. Conversely, in the open conformer, both HOMO-1 and LUMO + 6 in the open conformer have p*_x_* (first Te nucleus), p*_y_* (second Te nucleus) and p*_y_* (first Te nucleus), p*_x_* (second Te nucleus) character, so that magnetic coupling occurs simultaneously at both Te nuclei.

**Figure 6:**
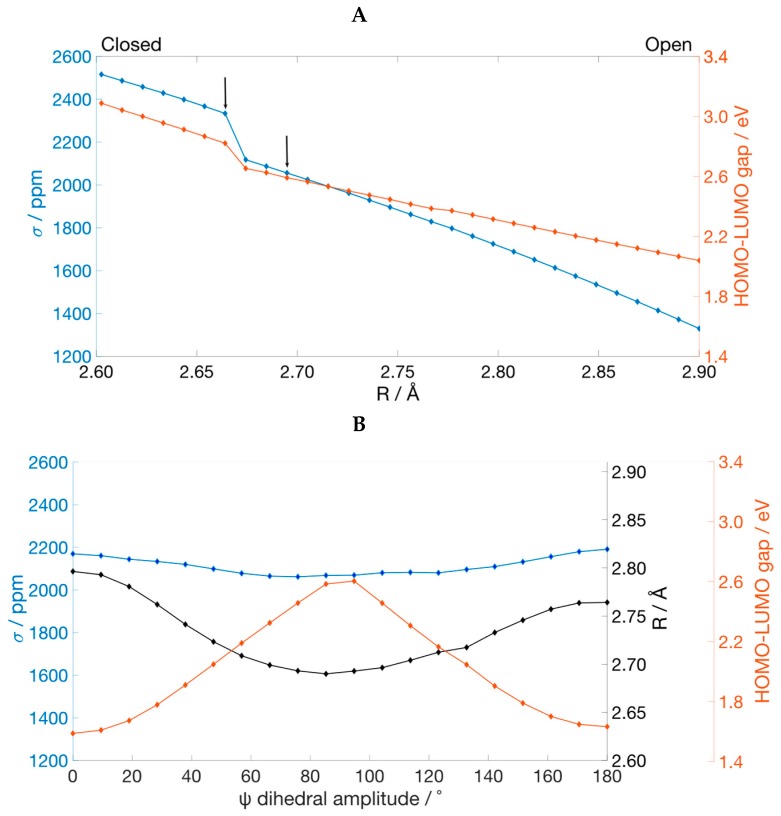
(**A**) Values of the ^125^Te isotropic shielding constant and of the HOMO-LUMO gap of Ph_2_Te_2_ at different Te-Te distances. The two arrows indicate the points at which the two fully optimized closed and open conformers are located. (**B**) Values of the ^125^Te isotropic shielding constant, of the Te-Te distance (R) and of the HOMO-LUMO gap of Ph_2_Te_2_ at different values of the Ψ dihedral angle. Level of theory: ZORA-OPBE/QZ4Pae//ZORA-OPBE/TZ2P.

The deformation of the Te–Te bond in Ph_2_Te_2_ has a strong effect on the σ(^125^Te). From the values of 2095.4 and 2390.2 ppm calculated for the two minima, we computed a value of 2515.5 ppm when the bond is compressed to 2.60 Å and of 1329.2 ppm when the bond length is increased from the equilibrium geometries to 2.90 Å. The σ(^125^Te) profile along the stretching of the Te–Te bond shows an almost linear decrease with the bond length (Figure 6A). Importantly, starting from the closed conformer, as the bond lengthens, a rotation of the phenyl groups occurs, resulting in the switch of the structure from closed to open after 2.66 Å. This exactly corresponds to the sudden drop of the shielding constant in Figure 6A. 

**Figure 7:**
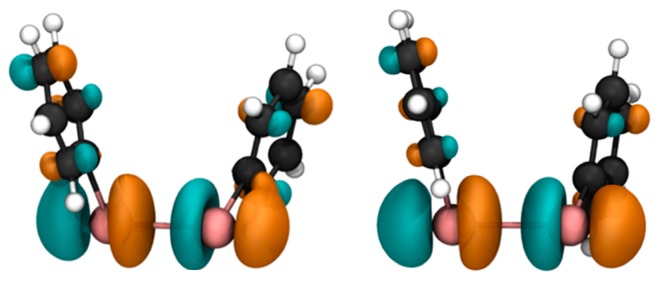
LUMO of Ph_2_Te_2_: relaxed geometry (**left**) and geometry with the maximum imposed Te–Te bond deformation (**right**). Isodensity 0.05 a.u. Level of theory: ZORA-OPBE/QZ4Pae//ZORA-OPBE/TZ2P.

The decomposition of σ(^125^Te) computed for the structure with the largest Te–Te distance shows that the value of σ_p_ changes from −3433.1 ppm in the minimum energy structure of the open conformer to −4054.5 ppm. Inspection of the most important contributions of σ_p_^occ-virt^ in the deformed geometry shows that the pairs of orbitals that play an important part in determining the value of the ^125^Te paramagnetic shielding constant involve the LUMO as the empty orbital (Table S4). The LUMO of Ph_2_Te_2_ is an anti-bonding σ MO mainly composed by the out-of-phase overlap of two p*_z_* orbitals on the two chalcogen atoms. Upon stretching of the Te–Te bond, it remains almost unchanged (Figure 7), but decreases in energy leading to a progressively smaller HOMO-LUMO gap (Figure 6A). Thus, since the σ_p_^occ-virt^ are weighted by a factor that is inversely proportional to the energy gap between the involved MOs, the stabilization of the LUMO implies an increase of the magnetic coupling of all pairs of MOs including the LUMO as the empty molecular orbital. This leads to a larger (negative) σ_p_ and consequently to a higher ^125^Te chemical shift. 

Finally, we investigated the effects of a rotation around the Te-Te bond on the ^125^Te shielding constant. Results showed a decrease of σ(^125^Te) from an initial value of 2169.3 ppm (Ψ = 0°) to a relative minimum of 2061.7 ppm recorded at Ψ = 76°, followed by an increase up to 2190.8 ppm (Figure 6B). Right after the minimum point, a non-monotonous increase can be observed, which is caused by the slightly different values of Φ_1_ and Φ_2_ at each point during the completely relaxed torsional scan. Nevertheless, the profile of σ(^125^Te) follows the trend of the Te–Te distance, since when the chalcogen atoms get closer or further, the shielding constant decreases or increases, respectively (Figure 6B). This behavior follows from Schreckenbach analysis since the elongation of the inter-chalcogen bond leads to a stabilization of the LUMO and thus to a larger (negative) σ_p_ and consequently to a higher ^125^Te chemical shift, as explained in the previous paragraph.

**Figure 8:**
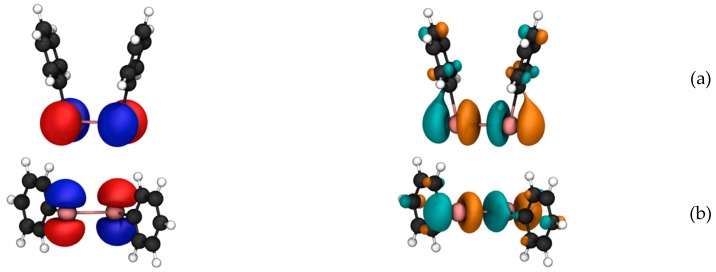
HOMO (left) and LUMO (right) of Ph_2_Te_2_ with Ψ = 0° (**a**) and with Ψ = 180° (**b**). Isodensity 0.05 a.u. Level of theory: ZORA-OPBE/QZ4Pae//ZORA-OPBE/TZ2P.

The HOMO–LUMO pair provides a very small σ_p_^occ-virt^ contribution in the structures with Ψ equal to 0° or 180° due to their shape (Figure 8) that results in almost no HOMO–LUMO overlap after application of the magnetic moment operator due to the cancellation of the positive and negative orbital phase. Conversely, the largest contributions to σ_p_ arise from the HOMO-2–LUMO and HOMO-1–LUMO pairs when Ψ = 0° with values of −716.8 and −698.1 ppm respectively, and from the pair HOMO-1–LUMO when Ψ = 180°, with a value of −801.7 ppm. Although the energy gap is larger than for HOMO–LUMO, the magnetic coupling is strong due to the composition of these orbital pairs in terms of Te p atomic orbitals. Comparing the two extreme structures obtained in the torsional scan with the fully optimized open conformer of Ph_2_Te_2_, it is clear how the intermediate value of Ψ found in the minimum energy structure, with the two phenyl rings arranged in skewed fashion, favors not only the strongest orbital interactions, but also enhances the magnetic couplings, resulting in the structure with the lowest isotropic shielding constant. These analyses have been carried out also with the inclusion of spin-orbit effects: the two limit structures, Ψ = 0° and Ψ = 180°, have been chosen because of the biggest difference between relativistic scalar and spin-orbit ^125^Te shielding constants. The larger, and therefore the main, σ_p_ contributions are localized as the same shown above using the scalar ZORA approximation (Tables S5 and S6). This allowed us to entirely perform the analysis within a scalar approximation without losing important magnetic coupling contributions and obtaining reliable data that are qualitatively comparable with those obtained with the extended spin-orbit Hamiltonian.

## 3. Materials and Methods 

All geometry optimizations were carried out with the Amsterdam density functional (ADF) program (version 2017, SCM, Amsterdam, The Netherlands) developed by Baerends and co-workers [47,48,49]. In agreement with a recent benchmark study [10], the OPBE functional [50,51,52,53] was chosen in combination with the TZ2P basis set, which is a large and uncontracted set of Slater-type orbitals (STOs) of triple-ζ quality, augmented with two sets of polarizations functions for each atom: 2p and 3d for hydrogen, 3d and 4f in the case of carbon, and 5d and 4f for tellurium. The frozen core approximation was employed: up to 1s for carbon and up to 4p for tellurium. Relativistic effects were included in the calculations using the scalar relativistic zeroth-order regular approximation (ZORA) [54]. This level of theory is denoted ZORA-OPBE/TZ2P. The scans of selected geometrical parameters were carried out at the ZORA-OPBE/TZ2P level of theory with the (linear) transit protocol implemented in ADF.

For the computation of the δ(^125^Te), the calculations of the shielding tensors were carried out with the NMR module of the ADF program (version 2017, SCM, Amsterdam, The Netherlands) [45], using the OPBE functional in combination with the QZ4P all-electron basis set [55] in chloroform (ε = 4.8) employing the COSMO solvation model [56,57]. This level of theory, which gave very good results for the δ(^77^Se) calculations on organic mono and diselenides [11], is here denoted COSMO-ZORA-OPBE/QZ4Pae. As a reference, in order to convert the isotropic shielding constant to δ(^125^Te)*,* using the relationship *δ* = σ_ref_ *−* σ*,* (CH_3_)_2_Te was employed [43] with a calculated σ_ref_ of 2670.3 ppm at the COSMO-ZORA-OPBE/QZ4Pae level of theory. Spin-orbit effects have been taken into account in selected cases and are denoted in this paper as COSMO-SO-ZORA-OPBE/QZ4Pae.

**Scheme 2:**
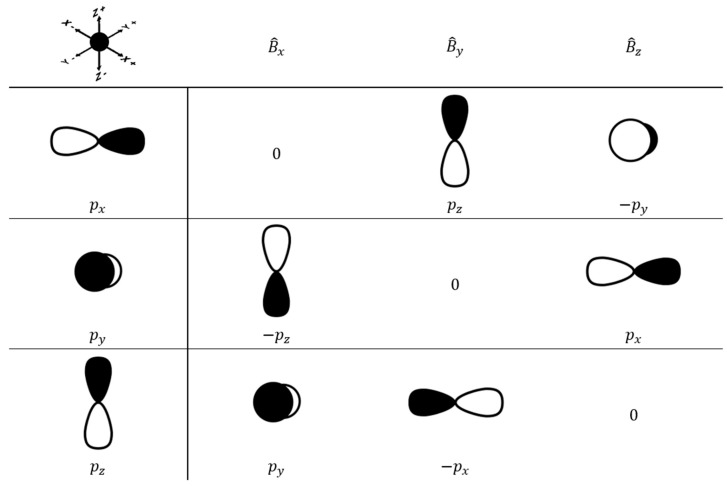
Transformations of *p* atomic orbitals due to magnetic field operator $\hat{B}$, which is proportional to the angular momentum operator [58,59]. In a minimal basis of AOs, the result of the action of the B-field quantum mechanics operator is a rotation of the original orbital (the factor −iℏ/2c is omitted).

The analysis of σ was carried out in the gas phase at the ZORA-OPBE/QZ4Pae level of theory. The shielding constant can be decomposed as the sum of the diamagnetic (σ_d_) and the paramagnetic (σ_p_) contributions [46]. The spin-orbit term was neglected because it was not explicitly considered in these calculations. The diamagnetic term depends mainly on the core–shell electrons and is therefore almost constant for a given nucleus in different compounds and cancels out when calculating chemical shifts. The paramagnetic term can be broken down into the sum of two contributions

(1) $$\sigma_{p}=\sigma_{p}^{\mathrm{occ}-\mathrm{occ}}+\sigma_{p}^{\mathrm{occ}-\mathrm{virt}},$$

in which σ_p_^occ-occ^ is a sum over all pairs of occupied orbitals and σ_p_^occ-virt^ is a sum over all pairs of orbitals consisting of an occupied orbital and virtual orbital; each term is weighted by the inverse of the energy gap and is proportional to the magnetic coupling between the involved molecular orbitals

(2) $$\langle \phi_{\mathrm{virt}}\left|\hat{B}\right|\phi_{\mathrm{occ}}\rangle ,$$

in which $\hat{B}$ is the magnetic moment operator (Scheme 2). σ_p_^occ-virt^ is the largest contribution to σ_p_ and is mostly responsible for the variation in chemical shift.

To run MD simulations, a novel set of GAFF (generalized AMBER force field) parameters for Ph_2_Te_2_ was obtained. A recent computational protocol devised by some of us was employed for this purpose [28]. The two energetically relevant conformers of Ph_2_Te_2_, fully optimized using OPBE [50,51,52,53] combined with the TZ2P basis set for all the atoms with the ADF program, were used as starting structures for all the scans (see below). Charges were calculated using R.E.D. Server Development [60] which is a web-based interface that employs PyRED program (version SEP-2015, Université de Picardie-Jules Verne, Amiens, France and Sanford Burnham Prebys Medical Discovery Institute, La Jolla, USA) [61] and R.E.D. tools (version SEP-2015, Université de Picardie-Jules Verne, Amiens, France and Sanford Burnham Prebys Medical Discovery Institute, La Jolla CA, USA) [62]. Calculations of the charges were carried out, as recommended [63], at the Hartree-Fock level of theory using the 6-31G(d) basis set for all the atoms except tellurium for which the Stuttgart Dresden effective core potential basis set [64,65] was employed; the Gaussian09 package (rev D.01, Gaussian, Inc.: Wallingford CT, USA) was used for these calculations [66]. To obtain highly reproducible charges, two different orientations were taken into account for each conformer, using the rigid-body re-orientation algorithm implemented in R.E.D. Tools. In order to obtain the final charge values for Ph_2_Te_2_, a simultaneous restrained electrostatic potential (RESP) [67] fit of four structures was performed. 

Constrained scans (i.e., calculations where only one relevant parameter is changed and the rest of the molecule is kept frozen) were carried out at ZORA-OPBE/TZ2P to obtain the force constants of the harmonic contributions (i.e., *a_i_* and *b_i_* terms in Equation S1). Stretching and bending parameters were thus computed for Te–C and Te–Te bonds and for Te–Te–C and Te–C–C angles, respectively. Relaxed scans of the most stable conformer were performed for the dihedrals C–Te–Te–C and C–C–Te–Te. Moreover, although generalized parameters for the dihedral Te–C–C–H (in which the chalcogen atom is in a terminal position) were already present in GAFF, we decided to improve the data by refitting the coefficients. The energies of the resulting ensemble of 72 structures were simultaneously fit with Paramfit [68], a tool included in AmberTools15 software [69]. These new parameters, which are included in the Supplementary Materials, were used in MD simulations of a single molecule of Ph_2_Te_2_ using the Assisted Model Building with Energy Refinement (AMBER) suite of programs (version 2015, University of California, San Francisco, San Francisco, CA, USA) [69]. The molecule was initially solvated in chloroform in a cubic box with a surrounding buffer of 12.0 Å. First, the potential energy was minimized by keeping the diphenyl ditelluride atoms fixed and relaxing the solvent; afterwards, a full minimization was done. The temperature was first increased using the Langevin thermostat up to 100 K for 20 ps and, subsequently, the Berendsen barostat was added for 1 ns to reach the production temperature (310 K) and pressure (1 bar). A pre-equilibration (200 ns) and production dynamics (500 ns) were conducted by switching to the Monte Carlo barostat. The cutoff distance for the nonbonded interactions was set to 10.0 Å; the shake constraints were applied to all hydrogen atoms, and a time step of 2 fs was used. 

## 4. Conclusions

In this work, the effects of structural changes on the ^125^Te chemical shift of Ph_2_Te_2_ were investigated using a combined quantum mechanical and classical computational methodology. Relativistic density functional theory-based ^125^Te-NMR calculations were performed using fully optimized conformers of Ph_2_Te_2_ as well as on structures extracted from a classical MD trajectory. At the scalar relativistic level, the agreement with the experimental result is satisfactory in both instances, but a non-negligible discrepancy can be observed. However, the inclusion of spin-orbit corrections leads to a much better agreement already when considering only the two energy-minimized open and closed conformers; the calculated result approaches the experimental values within just 17 ppm when the average geometry obtained from the dynamics is considered. Nonetheless, the MD simulation highlighted the high flexibility of Ph_2_Te_2_ molecule, affording a very wide span of calculated chemical shifts, from −225.5 to 1503.4 ppm as a function of the phenyl rings orientation and Te–Te bond lengths (see Figure S1 in Supplementary Materials). Again, the time-averaged ^125^Te chemical shift is, therefore, strongly dependent on the very accurate modeling of the whole potential energy surface, which suffers from the intrinsic approximations of classical force fields. Thus, the take-home message here is that conformational effects in diphenyl ditelluride (and closely related derivatives), are dominant over other issues like solvent, temperature, and aggregation, in the calculations of NMR properties. Carefully taking into account the conformational landscape and the spin-orbit correction leads to a very satisfactory agreement. 

Finally, a detailed decomposition of the calculated isotropic shielding constant was performed on the stable minima as well as on geometrically ad hoc deformed Ph_2_Te_2_ molecules to gain insight on the origin of the strong variation of ^125^Te chemical shift. Calculations on a simpler model monochalcogenide—i.e., PhTeH—were also included to derive more general conclusions about the relationship between the geometry and the shielding constant in organotellurides. 

The analyses conducted on PhTeH and Ph_2_Te_2_ show that, in general, a stretching of the bond in which Te is involved implies a lowering of the LUMO energy, a larger (negative) σ_p_, and consequently a higher ^125^Te chemical shift. Conversely, the rotation about a bond in which Te is involved—i.e., about the Te–Te bond (in Ph_2_Te_2_ ) or the C–Te bond (in PhTeH)—leads to larger (negative) σ_p_, whenever a favorable magnetic coupling of occupied/empty molecular orbital pairs is established; since in general these interactions involve high energy unoccupied molecular orbitals, the variation of ^125^Te σ (and δ) is less significant, due to the large energy gaps of these pairs.

## Figures and Tables

**Table 1:** Relative energies of Ph_2_Te_2_ conformers (kcal mol^−1^), Te–Te bond lengths (R, Å) and amplitudes of the Ψ and Φ_1,2_ dihedral angles (°) calculated with 23 different functionals combined with the TZ2P basis set; scalar ZORA effects were included in the calculations. Open conformer is taken as reference in the ΔE calculation.

Functional	R	Ψ	Φ_1,2_	R	Ψ	Φ_1,2_	ΔE
LDA	2.69	−79	81	2.65	−82	17	−2.1
BLYP	2.78	−87	97	2.74	−93	17	0.7
BP86	2.74	−86	98	2.70	−90	11	-^a^
OLYP	2.73	−87	98	2.70	−91	15	1.0
PBE	2.73	−85	93	2.70	−89	15	−0.4
OPBE	2.69	−87	100	2.66	−89	10	0.5
RPBE	2.75	−86	95	2.71	−90	15	0.5
revPBE	2.74	−86	97	2.71	−90	14	0.4
HTBS	2.71	−86	97	2.67	−89	10	0.1
PW91	2.73	−86	89	2.69	−88	13	−0.3
mPBE	2.74	−85	94	2.70	−89	15	−0.1
mPW	2.74	−86	98	2.70	−89	11	- ^a^
TPSS	2.73	−85	95	2.69	−90	16	0.0
M06-L	2.76	−60	96	2.70	−83	19	−1.5
B3LYP	2.74	−90	93	2.71	−97	27	0.9
PBE0	2.70	−88	90	2.67	−93	21	0.1
OPBE0	2.67	−89	96	2.64	−92	15	0.6
mPW1PW	2.71	−90	91	2.68	−94	23	-^a^
M06	2.73	−79	94	2.70	−93	26	-^a^
TPSSH	2.71	−86	94	2.68	−91	19	−0.3
BLYP-D3(BJ)	2.77	−69	89	2.70	−90	25	0.1
BP86-D3(BJ)	2.74	−60	95	2.69	−82	17	−0.1
PBE-D3(BJ)	2.73	−77	87	2.69	−84	17	−0.6

^a^ The difference is smaller than 0.1 kcal mol^−1^.

## Supplementary Materials

The following are available online: Figure S1: Values of the isotropic shielding constant of ^125^Te in the isolated Ph_2_Te_2_ molecule calculated at different times of the MD simulation; Table S1: Comparison between DFT and MM geometries in vacuo. Te–Te distance (R, Å), and relevant dihedrals (Ψ, Φ_1,2_, °); Table S2: Analysis of the ^125^Te paramagnetic contribution to the shielding constant (σ_p_) of PhTeH at the maximum imposed elongation of the Te–H bond; Table S3: Analysis of the paramagnetic shielding constant of Te in PhTeH after rotation of the C–C–Te–H dihedral of 90°; Table S4: Analysis of the ^125^Te paramagnetic contribution to the shielding constant (σ_p_) of Ph_2_Te_2_ at the maximum imposed elongation of the Te–Te bond; Table S5: Analysis of the ^125^Te paramagnetic contribution to the shielding constant (σ_p_) of Ph_2_Te_2_ with an imposed Ψ dihedral value of 0°; Table S6: Analysis of the ^125^Te paramagnetic contribution to the shielding constant (σ_p_) of Ph_2_Te_2_ with an imposed Ψ dihedral value of 180°; Table S7: Coordinates of the optimized conformers of Ph_2_Te_2_; Table S8: Coordinates of Ph_2_Te_2_ at the maximum elongation of the Te–Te bond; Table S9: Coordinates of Ph_2_Te_2_ with a Ψ dihedral value of 0°; Table S10: Coordinates of Ph_2_Te_2_ with a Ψ dihedral value of 180°; Table S11: Coordinates of the optimized structure of PhTeH; Table S12: Coordinates of PhTeH at the maximum elongation of the Te–H bond; Table S13: Coordinates of PhTeH after rotation of the C–C–Te–H dihedral of 90°; Equation S1: AMBER force field used in the MD simulations; Table S14: New GAFF parameters for Ph_2_Te_2_ molecule.

## References

[1] Krafft F., Lyons R.E. (1894). Ueber Diphenyltellurid und ein Verfahren zur Darstellung von Sulfidon, Seleniden und Telluriden. Ber. Dtsch. Chem. Ges..

[2] Zeiser F. (1895). Ueber einige schwefel-, selen- und tellurhaltige Ditolylverbindungen. Ber. Dtsch. Chem. Ges..

[3] Lederer K. (1915). Einwirkung von Phenylmagnesiumbromid auf Tellurdihalogene. Ber. Dtsch. Chem. Ges..

[4] Engman L. (1985). Synthetic applications of organotellurium chemistry. Acc. Chem. Res..

[5] Comasseto J.V. (2010). Selenium and tellurium chemistry: Historical background. J. Braz. Chem. Soc..

[6] Petragnani N., Comasseto J.V. (1991). Tellurium Reagents in Organic Synthesis; Recent Advances. Part 2. Synthesis.

[7] Zeni G., Lüdtke D.S., Panatieri R.B., Braga A.L. (2006). Vinylic Tellurides:  From Preparation to Their Applicability in Organic Synthesis. Chem. Rev..

[8] Petragnani N., Stefani H.A. (2005). Advances in organic tellurium chemistry. Tetrahedron.

[9] Kambe N., Fujioka T., Ogawa A., Miyoshi N., Sonoda N. (1988). Catalytic oxidation of olefins using diphenyl ditelluride. Phosphorus Sulfur Relat. Elem..

[10] Zaccaria F., Wolters L.P., Fonseca Guerra C.C., Orian L. (2016). Insights on selenium and tellurium diaryldichalcogenides: A benchmark DFT study. J. Comput. Chem..

[11] Ribaudo G., Bellanda M., Menegazzo I., Wolters L.P., Bortoli M., Ferrer-Sueta G., Zagotto G., Orian L. (2017). Mechanistic insight into the oxidation of organic phenylselenides by H_2_O_2_. Chem. Eur. J..

[12] Bortoli M., Zaccaria F., Dalla Tiezza M., Bruschi M., Fonseca Guerra C., Bickelhaupt F.M., Orian L. (2018). Oxidation of organic diselenides and ditellurides by H_2_O_2_ for bioinspired catalyst design. Phys. Chem. Chem. Phys..

[13] Orian L., Toppo S. (2014). Organochalcogen peroxidase mimetics as potential drugs: A long story of a promise still unfulfilled. Free Radic. Biol. Med..

[14] Dalla Tiezza M., Ribaudo G., Orian L. (2018). Organodiselenides: Organic Catalysis and Drug Design Learning from Glutathione Peroxidase. Curr. Org. Chem..

[15] Ibrahim M., Hassan W., Meinerz D.F., dos Santos M., Klimaczewski C.V., Deobald A.M., Costa M.S., Nogueira C.W., Barbosa N.B.V., Rocha J.B.T. (2012). Antioxidant properties of diorganoyl diselenides and ditellurides: Modulation by organic aryl or naphthyl moiety. Mol. Cell. Biochem..

[16] Meinerz D.F., Allebrandt J., Mariano D.O., Waczuk E.P., Soares F.A., Hassan W., Rocha J.B.T. (2014). Differential genotoxicity of diphenyl diselenide (PhSe)_2_ and diphenyl ditelluride (PhTe)_2_. PeerJ.

[17] Orian L., Mauri P., Roveri A., Toppo S., Benazzi L., Bosello-Travain V., De Palma A., Maiorino M., Miotto G., Zaccarin M. (2015). Selenocysteine oxidation in glutathione peroxidase catalysis: An MS-supported quantum mechanics study. Free Radic. Biol. Med..

[18] Orian L., Cozza G., Maiorino M., Toppo S., Ursini F., Flohé L. (2018). The mechanism of glutathione peroxidases. Glutathione.

[19] Bortoli M., Torsello M., Bickelhaupt F.M., Orian L. (2017). Role of the Chalcogen (S, Se, Te) in the Oxidation Mechanism of the Glutathione Peroxidase Active Site. ChemPhysChem.

[20] Heimfarth L., da Silva Ferreira F., Pierozan P., Mingori M.R., Moreira J.C.F., da Rocha J.B.T., Pessoa-Pureur R. (2017). Astrocyte-neuron interaction in diphenyl ditelluride toxicity directed to the cytoskeleton. Toxicology.

[21] Heimfarth L., da Silva Ferreira F., Pierozan P., Loureiro S.O., Mingori M.R., Moreira J.C.F., da Rocha J.B.T., Pessoa-Pureur R. (2016). Calcium signaling mechanisms disrupt the cytoskeleton of primary astrocytes and neurons exposed to diphenylditelluride. Biochim. Biophys. Acta Gen. Subj..

[22] da Luz S.C.A., Daubermann M.F., Thomé G.R., Dos Santos M.M., Ramos A., Torres Salazar G., da Rocha J.B.T., Barbosa N.V. (2015). Diphenyl ditelluride intoxication triggers histological changes in liver, kidney, and lung of mice. Anal. Cell. Pathol..

[23] Caeran Bueno D., Meinerz D.F., Allebrandt J., Waczuk E.P., dos Santos D.B., Mariano D.O.C., Rocha J.B.T. (2013). Cytotoxicity and genotoxicity evaluation of organochalcogens in human leucocytes: A comparative study between ebselen, diphenyl diselenide, and diphenyl ditelluride. Biomed Res. Int..

[24] Santos D.B., Schiar V.P.P., Paixão M.W., Meinerz D.F., Nogueira C.W., Aschner M., Rocha J.B.T., Barbosa N.B. (2009). Hemolytic and genotoxic evaluation of organochalcogens in human blood cells in vitro. Toxicol. Vitr..

[25] Nogueira C.W., Zeni G., Rocha J.B.T. (2004). Organoselenium and organotellurium compounds: Toxicology and pharmacology. Chem. Rev..

[26] Fuller A.L., Scott-Hayward L.A.S., Li Y., Bühl M., Slawin A.M.Z., Woollins J.D. (2010). Automated Chemical Crystallography. J. Am. Chem. Soc..

[27] Baldo M., Forchioni A., Irgolic K.J., Pappalardo G.C. (1978). Carbon-13 spin-lattice relaxation and molecular motion of diphenyl dichalcogenides. J. Am. Chem. Soc..

[28] Torsello M., Pimenta A.C., Wolters L.P., Moreira I.S., Orian L., Polimeno A. (2016). General AMBER Force Field Parameters for Diphenyl Diselenides and Diphenyl Ditellurides. J. Phys. Chem. A.

[29] Mardirossian N., Head-Gordon M. (2017). Thirty years of density functional theory in computational chemistry: An overview and extensive assessment of 200 density functionals. Mol. Phys..

[30] Barone V. (2016). The virtual multifrequency spectrometer: A new paradigm for spectroscopy. Wiley Interdiscip. Rev. Comput. Mol. Sci..

[31] Bagno A., Saielli G. (2015). Addressing the stereochemistry of complex organic molecules by density functional theory-NMR. Wiley Interdiscip. Rev. Comput. Mol. Sci..

[32] Grimblat N., Zanardi M.M., Sarotti A.M. (2015). Beyond DP4: An Improved Probability for the Stereochemical Assignment of Isomeric Compounds using Quantum Chemical Calculations of NMR Shifts. J. Org. Chem..

[33] Zanardi M.M., Biglione F.A., Sortino M.A., Sarotti A.M. (2018). General Quantum-Based NMR Method for the Assignment of Absolute Configuration by Single or Double Derivatization: Scope and Limitations. J. Org. Chem..

[34] Casella G., Bagno A., Komorovsky S., Repisky M., Saielli G. (2015). Four-Component Relativistic DFT Calculations of 13C Chemical Shifts of Halogenated Natural Substances. Chem. A Eur. J..

[35] Kaupp M., Malkina O.L., Malkin V.G., Pyykkö P. (1998). How Do Spin–Orbit-Induced Heavy-Atom Effects on NMR Chemical Shifts Function? Validation of a Simple Analogy to Spin–Spin Coupling by Density Functional Theory (DFT) Calculations on Some Iodo Compounds. Chem. A Eur. J..

[36] Rusakov Y.Y., Rusakova I.L., Krivdin L.B. (2016). On the HALA effect in the NMR carbon shielding constants of the compounds containing heavy p-elements. Int. J. Quantum Chem..

[37] Autschbach J., Zheng S., Webb A.G. (2009). Chapter 1 Relativistic Computations of NMR Parameters from First Principles: Theory and Applications.

[38] Rusakov Y.Y., Rusakova I.L. (2019). Long-range relativistic heavy atom effect on ^1^H NMR chemical shifts of selenium- and tellurium-containing compounds. Int. J. Quantum Chem..

[39] Jaszuński M., Olejniczak M. (2013). NMR shielding constants in SeH_2_ and TeH_2_. Mol. Phys..

[40] Ruiz-Morales Y., Schreckenbach G., Ziegler T. (1997). Calculation of ^125^Te Chemical Shifts Using Gauge-Including Atomic Orbitals and Density Functional Theory. J. Phys. Chem. A.

[41] Rusakova I.L., Rusakov Y.Y., Krivdin L.B. (2017). Calculation of ^125^Te NMR Chemical Shifts at the Full Four-Component Relativistic Level with Taking into Account Solvent and Vibrational Corrections: A Gateway to Better Agreement with Experiment. J. Phys. Chem. A.

[42] Elder P.J.W., Vargas-Baca I. (2016). ^125^Te NMR provides evidence of autoassociation of organo-ditellurides in solution. Phys. Chem. Chem. Phys..

[43] Granger P., Chapelle S., McWhinnie W.R., Al-Rubaie A. (1981). A ^125^Te NMR study of the exchange reaction between diarylditellurides. J. Organomet. Chem..

[44] Alkan F., Dybowski C. (2018). Spin-orbit effects on the ^125^Te magnetic-shielding tensor: A cluster-based ZORA/DFT investigation. Solid State Nucl. Magn. Reson..

[45] Schreckenbach G., Ziegler T. (1995). Calculation of NMR shielding tensors using gauge-including atomic orbitals and modern density functional theory. J. Phys. Chem..

[46] Schreckenbach G. (1999). The ^57^Fe nuclear magnetic resonance shielding in ferrocene revisited. A density-functional study of orbital energies, shielding mechanisms, and the influence of the exchange-correlation functional. J. Chem. Phys..

[47] te Velde G., Bickelhaupt F.M., Baerends E.J., Fonseca Guerra C., van Gisbergen S.J.A., Snijders J.G., Ziegler T. (2001). Chemistry with ADF. J. Comput. Chem..

[48] Guerra C.F., Snijders J.G., te Velde G., Baerends E.J. (1998). Towards an order-N DFT method. Theor. Chem. Acc..

[49] Baerends E.J., Ziegler T., Atkins A.J., Autschbach J., Bashford D., Baseggio O., Bérces A., Bickelhaupt F.M., Bo C., Boerritger P.M. (2017). ADF2017.

[50] Swart M., Ehlers A.W., Lammertsma K. (2004). Performance of the OPBE exchange-correlation functional. Mol. Phys..

[51] Handy N.C., Cohen A.J. (2001). Left-right correlation energy. Mol. Phys..

[52] Hoe W.-M., Cohen A.J., Handy N.C. (2001). Assessment of a new local exchange functional OPTX. Chem. Phys. Lett..

[53] Perdew J.P.J., Burke K., Ernzerhof M. (1996). Generalized Gradient Approximation Made Simple. Phys. Rev. Lett..

[54] van Lenthe E., Baerends E.J., Snijders J.G. (1994). Relativistic total energy using regular approximations. J. Chem. Phys..

[55] Van Lenthe E., Baerends E.J. (2003). Optimized Slater-type basis sets for the elements 1-118. J. Comput. Chem..

[56] Klamt A., Schüürmann G. (1993). COSMO: A new approach to dielectric screening in solvents with explicit expressions for the screening energy and its gradient. J. Chem. Soc. Perkin Trans..

[57] Pye C.C., Ziegler T. (1999). An implementation of the conductor-like screening model of solvation within the Amsterdam density functional package. Theor. Chem. Acc..

[58] Viesser R.V., Ducati L.C., Tormena C.F., Autschbach J. (2017). The unexpected roles of σ and π orbitals in electron donor and acceptor group effects on the ^13^C NMR chemical shifts in substituted benzenes. Chem. Sci..

[59] Autschbach J., Zheng S. (2008). Analyzing Pt chemical shifts calculated from relativistic density functional theory using localized orbitals: The role of Pt 5d lone pairs. Magn. Reson. Chem..

[60] Vanquelef E., Simon S., Marquant G., Garcia E., Klimerak G., Delepine J.C., Cieplak P., Dupradeau F.Y. (2011). R.E.D. Server: A web service for deriving RESP and ESP charges and building force field libraries for new molecules and molecular fragments. Nucleic Acids Res..

[61] Wang F., Becker J.P., Cieplak P., Dupradeau F.-Y. (2013). R.E.D. Python: Object Oriented Programming for AMBER Force Fields.

[62] Dupradeau F.Y., Pigache A., Zaffran T., Savineau C., Lelong R., Grivel N., Lelong D., Rosanski W., Cieplak P. (2010). The R.E.D. tools: Advances in RESP and ESP charge derivation and force field library building. Phys. Chem. Chem. Phys..

[63] Bayly C.C.I., Cieplak P., Cornell W.D., Kollmann P.A. (1993). A well-behaved electrostatic potential based method using charge restraints for deriving atomic charges: The RESP model. J. Phys. Chem..

[64] Igel-Mann G., Stoll H., Preuss H. (2006). Pseudopotentials for main group elements (IIIa through VIIa). Mol. Phys..

[65] Bergner A., Dolg M., Küchle W., Stoll H., Preuß H. (1993). Ab initio energy-adjusted pseudopotentials for elements of groups 13–17. Mol. Phys..

[66] Frisch M.J., Trucks G.W., Schlegel H.B., Scuseria G.E., Robb M.A., Cheeseman J.R., Scalmani G., Barone V., Mennucci B., Petersson G.A. (2009). Gaussian 09, Rev D.01.

[67] Cornell W.D., Cieplak P., Bayly C.I., Kollman P.A., Kollmann P.A. (1993). Application of RESP Charges To Calculate Conformational Energies, Hydrogen Bond Energies, and Free Energies of Solvation. J. Am. Chem. Soc..

[68] Betz R.M., Walker R.C. (2015). Paramfit: Automated optimization of force field parameters for molecular dynamics simulations. J. Comput. Chem..

[69] Case D.A., Berryman J.T., Betz R.M., Cerutti D.S., Cheatham III T.E., Darden T.A., Duke R.E., Giese T.J., Gohlke H., Goetz A.W. (2015). AMBER 2015.

