# Supplementary material for: The 125Te Chemical Shift of Diphenyl Ditelluride: Chasing Conformers over a Flat Energy Surface
Source: Molecules. 2019 Mar 30;24(7):1250. doi: 10.3390/molecules24071250 (PMC6480379; doi:10.3390/molecules24071250)
Supplement: Supplementary file 1 [file molecules-24-01250-s001.pdf]

# – SUPPLEMENTARY MATERIALS –

The ^{125}Te chemical shift of diphenyl ditelluride: chasing conformers over a flat energy surface

Marco Bortoli [1], Marco Dalla Tiezza [1], Cecilia Muraro [1], Giacomo Saielli [1,2] and Laura Orian [1,*]

[1] Dipartimento di Scienze Chimiche, Universita` degli Studi di Padova, Via Marzolo 1, 35131 Padova, Italy marco.bortoli@studenti.unipd.it; marco.dallatiezza@studenti.unipd.it; cecilia.muraro@studenti.unipd.it
[2] CNR Institute on Membrane Technology, Padova Unit, 35131 Padova, Italy; giacomo.saielli@unipd.it
* Correspondence: laura.orian@unipd.it; Tel.: +39-049-8275140

Table of contents

Figure S1 Values of the isotropic shielding constant of ^{125}Te in the isolated Ph_2Te_2 molecule calculated at different times of the MD simulation. 3

Table S1 Comparison between DFT and MM geometries *in vacuo*. Te-Te distance (R, Å), and relevant dihedrals (Ψ, $\Phi_{1,2}$, °). 3

Table S2 Analysis of the ^{125}Te paramagnetic contribution to the shielding constant (σ_P) of PhTeH [a] at the maximum imposed elongation of the Te-H bond 3

Table S4 Analysis of the ^{125}Te paramagnetic contribution to the shielding constant (σ_P) of Ph_2Te_2 [a] at the maximum imposed elongation of the Te-Te bond 4

Table S5 Analysis of the ^{125}Te paramagnetic contribution to the shielding constant (σ_P) of Ph_2Te_2 [a] with an imposed Ψ dihedral value of 0˚ 5

Table S6 Analysis of the ^{125}Te paramagnetic contribution to the shielding constant (σ_P) of Ph_2Te_2 [a] with an imposed Ψ dihedral value of 180˚ 5

Table S7 Coordinates of the optimized conformers of Ph_2Te_2. 6

Table S8 Coordinates of Ph_2Te_2 at the maximum elongation of the Te-Te bond. 6

Table S9 Coordinates of Ph_2Te_2 with a Ψ dihedral value of 0˚. 6

Table S10 Coordinates of Ph_2Te_2 with a Ψ dihedral value of 180˚. 7

Table S11 Coordinates of the optimized structure of PhTeH. 7

Table S12 Coordinates of PhTeH at the maximum elongation of the Te-H bond. 7

Table S13 Coordinates of PhTeH after rotation of the C-C-Te-H dihedral of 90°. 8

Table S14 Coordinates of the reference molecule Me_2Te. 8

Equation S1 AMBER force field used in the MD simulations 9

Table S15 New GAFF parameters for Ph_2Te_2 molecule. 9

Figure S2 Values of ^{125}Te chemical shift in the isolated Ph_2Te_2 molecule calculated with and without Spin-Orbit corrections during the rotation of Ψ dihedral. 9

Figure S3 HOMO-1 (left) and HOMO-2 (right) of Ph_2Te_2 with an imposed Ψ dihedral value of 0 ° 10

Figure S4 HOMO-1 orbital of Ph_2Te_2 with an imposed Ψ dihedral value of 180 ° 10

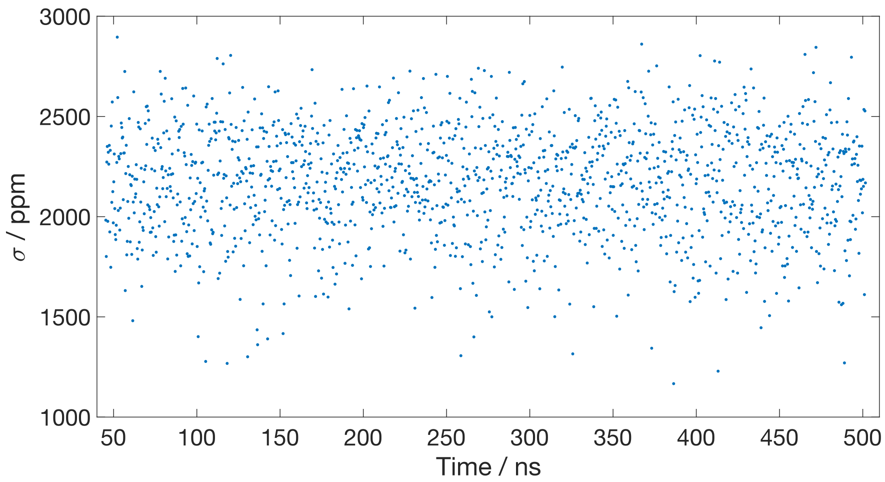

Figure S1 Values of the isotropic shielding constant of ^{125}Te in the isolated Ph_2Te_2 molecule calculated at different times of the MD simulation. Level of theory COSMO-ZORA-OPBE/QZ4Pae

Table S1 Comparison between DFT and MM geometries *in vacuo*. Te-Te distance (R, Å), and relevant dihedrals (Ψ, $\Phi_{1,2}$, °). Levels of theory: DFT ZORA-OPBE/TZ2P; MM AMBER-ff14SB

	DFT		MM
	Open	Closed	
R	2.69	2.66	2.68
Ψ	-87	-89	-82
Φ_1	100	10	16
Φ_2	100	10	16

Table S2 Analysis of the ^{125}Te paramagnetic contribution to the shielding constant (σ_P) of PhTeH [a] at the maximum imposed elongation of the Te-H bond: $\sigma_P^{(occ\text{-}virt)}$ terms (ppm) and energy difference between the two involved orbitals ($\Delta\varepsilon$, eV). Level of theory: ZORA-OPBE/QZ4Pae//ZORA-OPBE/TZ2P

occ	virt	$\sigma_P^{(occ\text{-}virt)}$	$\Delta\varepsilon$
HOMO – 7	LUMO + 9	-129.6	5.72
HOMO – 4	LUMO	-611.6	3.72
HOMO – 3	LUMO + 3	-112.0	6.34
HOMO – 1	LUMO + 3	-123.0	5.28
HOMO	LUMO	-4998.8	1.10
	LUMO + 2	-197.0	3.67
	LUMO + 3	-838.1	4.17

[a] Contributions that are smaller (absolute value) than 100 ppm are not shown.

Table S3 Analysis of the paramagnetic shielding constant of Te in PhTeH after rotation of the C-C-Te-H dihedral of 90°: [a] contribution of the orbital pair to the paramagnetic shielding constant ($\sigma_P^{(occ\text{-}virt)}$, ppm) and energy difference between the two involved orbitals ($\Delta\varepsilon^{(occ\text{-}virt)}$, eV)

Occ	Virt	$\sigma_P^{(occ\text{-}virt)}$	$\Delta\varepsilon$
HOMO – 3	LUMO + 2	-251.0	6.32
HOMO	LUMO	-288.4	3.45
	LUMO + 2	-774.0	3.98
	LUMO + 3	-316.6	4.58

Table S4 Analysis of the ^{125}Te paramagnetic contribution to the shielding constant (σ_P) of Ph_2Te_2 [a] at the maximum imposed elongation of the Te-Te bond: $\sigma_P^{(occ\text{-}virt)}$ terms (ppm) and energy difference between the two involved orbitals ($\Delta\varepsilon$, eV). Level of theory: ZORA-OPBE/QZ4Pae//ZORA-OPBE/TZ2P.

occ	virt	$\sigma_P^{(occ\text{-}virt)}$ [b]	$\Delta\varepsilon$
HOMO – 8	LUMO	224.1	4.91
	LUMO + 5	128.4	7.00
HOMO – 7	LUMO	-234.0	4.51
HOMO – 6	LUMO	-342.7	4.35
	LUMO + 5	-168.6	6.45
	LUMO + 6	-113.9	6.52
HOMO – 2	LUMO	-352.9	3.06
	LUMO + 5	-115.0	5.15
	LUMO + 6	-138.7	5.23
HOMO – 1	LUMO	-670.4	2.14
	LUMO + 6	-598.7	4.31
HOMO	LUMO	-636.4	2.06
	LUMO + 5	-199.3	4.15

[a] Contributions that are smaller (absolute value) than 100 ppm are not shown.

Table S5 Analysis of the ^{125}Te paramagnetic contribution to the shielding constant (σ_P) of Ph_2Te_2 [a] with an imposed Ψ dihedral value of 0°: $\sigma_P^{(occ\text{-}virt)}$ terms (ppm) and energy difference between the two involved orbitals (Δε, eV). Level of theory: (SO)-ZORA-OPBE/QZ4Pae//ZORA-OPBE/TZ2P

		Scalar		SO	
occ	virt	$\sigma_P^{(occ\text{-}virt)}$ [b]	Δε	$\sigma_P^{(occ\text{-}virt)}$ [b]	Δε
HOMO – 8	LUMO	232.8	5.34	270.5	5.37
HOMO – 7	LUMO	-319.0	4.82	-233.3	4.86
	LUMO + 7	-126.8	7.17	-179.9	7.22
	LUMO + 10	-102.5	7.75	-172.1	7.80
HOMO – 6	LUMO + 1	-132.7	5.68	-255.3	5.66
HOMO – 2	LUMO	-716.8	3.45	-1193.6	3.46
HOMO – 1	LUMO	-698.1	3.07	-602.3	3.07
	LUMO + 1	-355.3	4.19	-285.7	4.21
	LUMO + 5	-104.6	4.83	-64.0	4.84
HOMO	LUMO + 7	-280.1	3.95	-260.1	3.94
	LUMO + 10	-204.5	4.53	-187.0	4.52

[a] Contributions that are smaller (absolute value) than 100 ppm are not shown.

Table S6 Analysis of the ^{125}Te paramagnetic contribution to the shielding constant (σ_P) of Ph_2Te_2 [a] with an imposed Ψ dihedral value of 180°: $\sigma_P^{(occ\text{-}virt)}$ terms (ppm) and energy difference between the two involved orbitals (Δε, eV). Level of theory: (SO)-ZORA-OPBE/QZ4Pae//ZORA-OPBE/TZ2P

		Scalar		SO	
occ	virt	$\sigma_P^{(occ\text{-}virt)}$ [b]	Δε	$\sigma_P^{(occ\text{-}virt)}$ [b]	Δε
HOMO – 7	LUMO	-318.7	5.13	-352.5	5.15
	LUMO + 1	-141.9	6.33	-189.6	6.35
HOMO – 6	LUMO + 6	-250.8	6.55	-247.9	6.55
HOMO – 5	LUMO	-110.7	4.06	-126.6	4.06
HOMO – 1	LUMO	-801.7	3.16	-860.9	3.17
	LUMO + 1	-191.1	4.36	-201.9	4.37
	LUMO + 5	-220.0	4.99	-163.6	5.01
HOMO	LUMO + 3	-112.2	2.90	-109.2	2.89
	LUMO + 6	-456.5	3.72	-424.5	3.72
	LUMO + 7	-111.1	4.06	-103.0	4.04

[a] Contributions that are smaller (absolute value) than 100 ppm are not shown.

Table S7 Coordinates of the optimized conformers of Ph_2Te_2. Level of theory ZORA-OPBE/TZ2P

Open Conformer

E = -5.50273579 Ha

Te	-0.904891	-0.996129	-2.273711
Te	0.904899	0.996145	-2.273755
C	-0.135427	-2.273671	-0.760487
C	0.874878	-3.191409	-1.054436
C	1.338736	-4.055191	-0.067137
C	0.793460	-4.013510	1.212731
C	-0.217879	-3.104183	1.505320
C	-0.683119	-2.234021	0.523179
C	0.135449	2.273679	-0.760511
C	0.683108	2.233995	0.523168
C	0.217830	3.104119	1.505325
C	-0.793509	4.013445	1.212738
C	-1.338745	4.055168	-0.067145
C	-0.874852	3.191422	-1.054459
H	1.301416	-3.231390	-2.054330
H	2.128571	-4.767184	-0.302343
H	1.156509	-4.692327	1.982643
H	-0.648880	-3.067871	2.504859
H	-1.470946	-1.521767	0.758769
H	1.470924	1.521732	0.758764
H	0.648795	3.067770	2.504878
H	-1.156591	4.692229	1.982663
H	-2.128579	4.767163	-0.302350
H	-1.301366	3.231430	-2.054363

Closed Conformer

E = -5.50193037 Ha

Te	-0.886663	-0.991019	-2.222910
Te	0.886776	0.991036	-2.222788
C	-0.126436	-2.326579	-0.748450
C	-0.741336	-3.577755	-0.671742
C	-0.319375	-4.500074	0.280979
C	0.715756	-4.183671	1.154910
C	1.326466	-2.936461	1.071148
C	0.907651	-2.005920	0.124875
C	0.126355	2.326574	-0.748420
C	-0.906997	2.005434	0.125603
C	-1.325928	2.935957	1.071840
C	-0.716044	4.183620	1.154894
C	0.318359	4.500499	0.280276
C	0.740409	3.578209	-0.672434
H	-1.548692	-3.848119	-1.351720
H	-0.803627	-5.474145	0.333396
H	1.045876	-4.907410	1.897718
H	2.138011	-2.678329	1.750286
H	1.393673	-1.033913	0.065247
H	-1.392377	1.033075	0.066538
H	-2.136902	2.677451	1.751519
H	-1.046250	4.907342	1.897681
H	0.801945	5.474932	0.332123
H	1.547148	3.848960	-1.352988

Table S8 Coordinates of Ph_2Te_2 at the maximum elongation of the Te-Te bond. Level of theory ZORA-OPBE/TZ2P

E = -5.49320682 Ha

Te	-2.615889	-1.256666	-0.678084
Te	-1.505192	0.528420	-2.675537
C	-0.904266	-2.399165	-0.191988
C	-0.090708	-2.022898	0.880101
C	1.008323	-2.802560	1.227005
C	1.300978	-3.959037	0.510669
C	0.489717	-4.338191	-0.554574
C	-0.610934	-3.563644	-0.906226
C	-0.767426	2.072337	-1.432504
C	-1.613255	3.092356	-0.989208
C	-1.107456	4.126176	-0.208344
C	0.242791	4.154367	0.128354
C	1.088811	3.143947	-0.317394
C	0.587961	2.103421	-1.094023
H	-0.314418	-1.119142	1.442290
H	1.638806	-2.502520	2.062766
H	2.161978	-4.566751	0.783856
H	0.713033	-5.243339	-1.117377
H	-1.241965	-3.863439	-1.739733
H	-2.668411	3.077204	-1.252333
H	-1.773526	4.915760	0.136446
H	0.635968	4.966112	0.738074
H	2.146426	3.161431	-0.058094
H	1.251965	1.312228	-1.434818

Table S9 Coordinates of Ph_2Te_2 with a Ψ dihedral value of 0°. Level of theory ZORA-OPBE/TZ2P

E = -5.48611008 Ha

Te	-2.343364	-1.741854	-1.640357	C	1.749364	2.241909	-0.722118
Te	-2.053101	0.928766	-2.416997	C	0.772688	1.681572	-1.539696
C	-0.937057	-1.906984	-0.076615	H	0.730503	-2.300016	-1.386267
C	0.396890	-2.213221	-0.355297	H	2.334327	-2.650931	0.463321
C	1.295350	-2.413696	0.687219	H	1.573990	-2.481447	2.821568
C	0.869003	-2.318690	2.008128	H	-0.799808	-1.944460	3.319211
C	-0.460150	-2.018174	2.287249	H	-2.401738	-1.572560	1.470121
C	-1.363406	-1.808785	1.249733	H	-1.927390	2.110901	0.485414
C	-0.554412	1.629406	-1.106857	H	-0.182511	3.088870	1.939695
C	-0.894318	2.140107	0.148083	H	2.175806	3.183783	1.167095
C	0.088775	2.693002	0.962184	H	2.781283	2.282946	-1.067546
C	1.409909	2.746192	0.528977	H	1.040487	1.288814	-2.517595

Table S10 Coordinates of Ph_2Te_2 with a Ψ dihedral value of 180°. Level of theory ZORA-OPBE/TZ2P

E = -5.48963046 Ha

				C	-0.419282	3.090909	-4.370870
				C	-1.169860	2.326279	-3.482582
Te	-0.735887	-1.460567	-1.461132	H	-0.462238	-2.405939	1.509549
Te	-1.860278	0.981079	-0.817193	H	-1.792157	-3.765308	3.092357
C	-1.909146	-2.556481	-0.082512	H	-3.994237	-4.684281	2.401053
C	-1.425268	-2.807696	1.203434	H	-4.857550	-4.236252	0.115264
C	-2.175968	-3.572152	2.091778	H	-3.527044	-2.881923	-1.471116
C	-3.409182	-4.086507	1.704190	H	0.928388	2.407107	-0.805450
C	-3.893091	-3.835853	0.424014	H	2.258534	3.762056	-2.391617
C	-3.147200	-3.074086	-0.470460	H	1.397463	4.205831	-4.679087
C	-0.687175	2.077366	-2.195731	H	-0.802116	3.282201	-5.372177
C	0.549489	2.597512	-1.806799	H	-2.131908	1.922724	-3.7894
C	1.295196	3.359590	-2.701174				
C	0.812533	3.607885	-3.982268				

Table S11 Coordinates of the optimized structure of PhTeH. Level of theory ZORA-OPBE/TZ2P

E = -2.86286441 Ha

				C	0.908771	-0.960868	0.001322
				H	-2.229607	-2.284481	-0.002445
Te	-1.674219	0.741083	-0.000001	H	-0.932002	-4.366843	-0.002313
H	-0.367332	1.764608	0.000011	H	1.555907	-4.302459	0.000037
C	-0.487001	-0.997924	-0.000026	H	2.722471	-2.106015	0.002335
C	-1.141981	-2.231845	-0.001340	H	1.439333	-0.011663	0.002394
C	-0.407560	-3.412482	-0.001284				
C	0.982975	-3.377348	0.000027				
C	1.634142	-2.148105	0.001309				

Table S12 Coordinates of PhTeH at the maximum elongation of the Te-H bond. Level of theory ZORA-OPBE/TZ2P

E = -2.77736595 Ha

				C	-1.689343	-3.973380	-0.586487
				C	-0.818012	-2.914302	-0.360932
Te	0.000000	0.000000	0.000000	H	-3.078228	-0.366990	-0.304812
C	-1.313406	-1.608140	-0.259696	H	-4.622792	-2.259014	-0.710194
C	-2.689370	-1.379277	-0.386239	H	-3.736091	-4.571926	-0.888786
C	-3.554157	-2.443530	-0.612896	H	-1.298282	-4.986610	-0.663783
C	-3.056231	-3.739954	-0.712673	H	0.249350	-3.096721	-0.260969

H	0.000000	0.000000	2.660000

Table S13 Coordinates of PhTeH after rotation of the C-C-Te-H dihedral of 90°. Level of theory ZORA-OPBE/TZ2P

E = -2.86183405 Ha

Te	-1.674200	0.741100	0.000000
H	-1.733822	0.831688	-1.656436
C	-0.487000	-0.997900	0.000000
C	-1.142000	-2.231800	-0.001300
C	-0.407600	-3.412500	-0.001300
C	0.983000	-3.377300	0.000000
C	1.634100	-2.148100	0.001300
C	0.908800	-0.960900	0.001300
H	-2.229600	-2.284500	-0.002400
H	-0.932000	-4.366800	-0.002300
H	1.555900	-4.302500	0.000000
H	2.722500	-2.106000	0.002300
H	1.439300	-0.011700	0.002400

Table S14 Coordinates of the reference molecule Me_2Te. Level of theory ZORA-OPBE/TZ2P

E = -1.55519407 Ha

Te	0.000000	0.000000	0.000000
C	0.000000	0.000000	-2.164359
H	0.493166	0.899240	-2.534463
H	0.496429	-0.897632	-2.534291
H	-1.045253	-0.001967	-2.479153
C	2.155252	0.000000	0.197676
H	2.568981	-0.898284	-0.261719
H	2.568908	0.898620	-0.260950
H	2.373355	-0.000470	1.267237

Equation S1 AMBER force field used in the MD simulations

$$V_{AMBER} = \sum_{i<j}^{n'_{atoms}} \frac{q_i q_j}{4\pi\varepsilon_0 r_{ij}} + \sum_{i<j}^{n'_{atoms}} 4\epsilon_{ij}\left[\left(\frac{\sigma_{ij}}{r_{ij}}\right)^{12} - \left(\frac{\sigma_{ij}}{r_{ij}}\right)^{6}\right] +$$

$$+ \sum_{i}^{n_{bonds}} b_i\left(r_i - r_{i,eq}\right)^2 + \sum_{i}^{n_{angles}} a_i\left(\theta_i - \theta_{i,eq}\right)^2 + \sum_{i}^{n_{dihedrals}} \sum_{n}^{n_{i,max}} \frac{V_{i,n}}{2}\left[1 + \cos\left(n\phi_i - \gamma_{i,n}\right)\right]$$

Table S15 New GAFF parameters for Ph_2Te_2 molecule. Bond equilibrium distances (r_{eq}, Å), bond force constants (b, kcal mol^{-2} Å^{-2}), angle equilibrium amplitudes (θ_{eq}, °), angle force constants (kcal mol^{-2} rad^{-2}) and dihedral Fourier term coefficients (V_i, kcal mol^{-1})

Bond		Angle		Dihedral[a]			
r_{eq}	b	θ_{eq}	a	$V_1/2$	$V_2/2$	$V_3/2$	$V_4/2$
Te-Te		Te-Te-C		C-Te-Te-C			
2.68	56.6	103.1	45.2	1.775 (0.0)	2.673 (0.0)	0.691 (0.0)	0.105 (0.0)
Te-C		Te-C-C		Te-Te-C-C			
2.13	78.0	123.7	59.8	0.012 (180.0)	0.198 (180.0)	0.785 (180.0)	0.036 (180.0)
				Te-C-C-H			
				2.235 (180.0)			

[a] γ values for the corresponding term in parentheses in degrees.

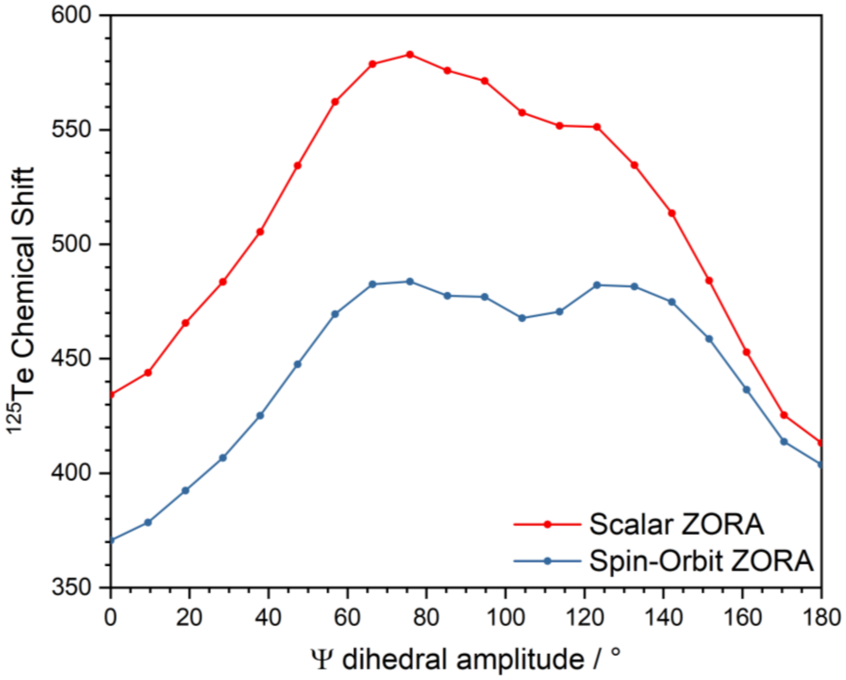

Figure S2 Values of ^{125}Te chemical shift in the isolated Ph_2Te_2 molecule calculated with and without Spin-Orbit corrections during the rotation of Ψ dihedral. Level of theory COSMO-(SO)-ZORA-OPBE/QZ4Pae

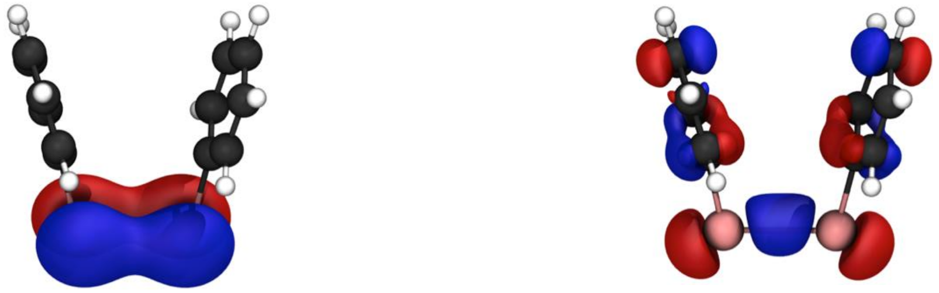

Figure S3 HOMO-1 (left) and HOMO-2 (right) of Ph_2Te_2 with an imposed Ψ dihedral value of 0 °. Level of theory ZORA-OPBE/TZ2P

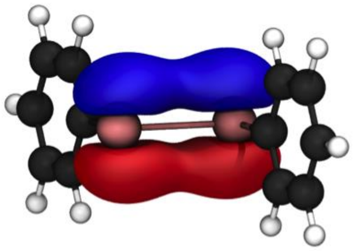

Figure S4 HOMO-1 orbital of Ph_2Te_2 with an imposed Ψ dihedral value of 180 °. Level of theory ZORA-OPBE/TZ2P
